# CD147 Promotes Entry of Pentamer-Expressing Human Cytomegalovirus into Epithelial and Endothelial Cells

**DOI:** 10.1128/mBio.00781-18

**Published:** 2018-05-08

**Authors:** Adam L. Vanarsdall, Sarah R. Pritchard, Todd W. Wisner, Jing Liu, Ted S. Jardetzky, David C. Johnson

**Affiliations:** a Department of Molecular Microbiology and Immunology, Oregon Health and Sciences University, Portland, Oregon, USA; b Department of Structural Biology, Stanford University School of Medicine, Stanford, California, USA; Princeton University

**Keywords:** HCMV entry, HCMV entry mediator, HCMV pentamer, HCMV trimer

## Abstract

Human cytomegalovirus (HCMV) replicates in many diverse cell types *in vivo*, and entry into different cells involves distinct entry mechanisms and different envelope glycoproteins. HCMV glycoprotein gB is thought to act as the virus fusogen, apparently after being triggered by different gH/gL proteins that bind distinct cellular receptors or entry mediators. A trimer of gH/gL/gO is required for entry into all cell types, and entry into fibroblasts involves trimer binding to platelet-derived growth factor receptor alpha (PDGFRα). HCMV entry into biologically relevant epithelial and endothelial cells and monocyte-macrophages also requires a pentamer, gH/gL complexed with UL128, UL130, and UL131, and there is evidence that the pentamer binds unidentified receptors. We screened an epithelial cell cDNA library and identified the cell surface protein CD147, which increased entry of pentamer-expressing HCMV into HeLa cells but not entry of HCMV that lacked the pentamer. A panel of CD147-specific monoclonal antibodies inhibited HCMV entry into epithelial and endothelial cells, but not entry into fibroblasts. shRNA silencing of CD147 in endothelial cells inhibited HCMV entry but not entry into fibroblasts. CD147 colocalized with HCMV particles on cell surfaces and in endosomes. CD147 also promoted cell-cell fusion induced by expression of pentamer and gB in epithelial cells. However, soluble CD147 did not block HCMV entry and trimer and pentamer did not bind directly to CD147, supporting the hypothesis that CD147 acts indirectly through other proteins. CD147 represents the first HCMV entry mediator that specifically functions to promote entry of pentamer-expressing HCMV into epithelial and endothelial cells.

## IMPORTANCE

Human cytomegalovirus infects nearly 80% of the world’s population and causes significant morbidity and mortality. The current method of treatment involves the use of antiviral agents that are prone to resistance and can be highly toxic to patients; currently, there is no vaccine against HCMV available. HCMV infections involve virus dissemination throughout the body, infecting a wide variety of tissues; however, the mechanism of spread is not well understood, particularly with regard to which cellular proteins are utilized by HCMV to establish infection. This report describes the characterization of a newly identified cellular molecule that affects HCMV entry into epithelial and endothelial cells. These results will lead to a better understanding of HCMV pathogenesis and have implications for the development of future therapeutics.

## INTRODUCTION

Human cytomegalovirus (HCMV) is a ubiquitous betaherpesvirus that establishes a lifelong persistent infection. Infection of a host who has a functioning immune system typically results in a mild fever or mononucleosis-like disease. In contrast, infection of an immunocompromised host can result in severe pneumonia, gastrointestinal disease, hepatitis, retinitis, and encephalitis (1, 2). HCMV infection can be particularly devastating to newborns and is the leading viral cause of birth defects, with 5 to 10% of congenitally infected children developing serious neurological defects, including hearing loss, mental retardation, and cerebral palsy (3, 4). HCMV is also a major problem in transplant patients due to immunosuppression and can exacerbate graft-versus-host disease and increase transplant vascular sclerosis (5).

Active HCMV infections frequently involve dissemination throughout the body, involving infection of many organs and different cell types (6–9). Epithelial cells, particularly in the retina and the gut, are highly susceptible to HCMV infection (10). Monocytes are thought to be the cells that harbor latent or persistent HCMV, and reactivation in monocyte-macrophages can lead to spread of HCMV in the blood followed by macrophage-mediated infection of endothelial cells and transmission into solid tissues (11).

All herpesviruses encode the viral fusion protein gB and different forms of the gH/gL proteins, which are thought to trigger the fusogenic activity of gB (12–14). HCMV gH/gL forms at least three protein complexes and perhaps four. gH/gL/gO is known as the trimer and involves assembly of gO onto gH/gL (15). The trimer is essential for entry into all cell types tested to date (16). The HCMV pentamer involves assembly of three smaller proteins: UL128, UL130, and UL131, all encoded in the ULb′ region of the virus genome, onto gH/gL (17–19). Passage of HCMV in human fibroblasts leads to loss of ULb′ genes (including the UL128 to -131 genes), which are important for virus replication in epithelial and endothelial cells and monocyte-macrophages (19, 20). Restoration of wild-type UL128 to -131 genes in HCMV lab strains allows repaired HCMV to infect these cell types (19). Defects in replication of HCMV lacking the pentamer involve the inability to enter endothelial and epithelial cells and monocyte-macrophages (21, 22). Our interference studies have shown that the trimer blocks HCMV entry into fibroblasts and the pentamer blocks entry into epithelial and endothelial cells (23, 24). Importantly, there was little or no interference when HCMV gH/gL or gB was expressed in any cell type. More recently, soluble forms of the trimer and pentameric complexes were also able to block entry of HCMV into fibroblasts and epithelial cells, respectively (25, 26). Together, these data strongly support the hypothesis that the trimer and pentamer mediate entry into different cell types by interacting with distinct cellular molecules. We recently described a third HCMV glycoprotein complex composed of gB and gH/gL that was found in virus-infected cells and in extracellular virions, although there is currently no evidence that this complex is essential for entry (27). There may also be gH/gL (free of these other proteins) in virions, but again the functional relevance of gH/gL is unclear.

Several cellular molecules have been described as receptors (molecules to which the virus binds) or entry mediators (a broader term that does not necessarily imply direct virus binding). The epidermal growth factor receptor (EGFR) was proposed to function as an HCMV receptor and it was reported that gB bound EGFR (28). Subsequent studies challenged the notion that EGFR is important for HCMV entry into fibroblast, epithelial, and endothelial cells, though EGFR signaling may enhance HCMV replication in some cells (29–32). The platelet-derived growth factor receptor alpha (PDGFRα) was also proposed to serve as an HCMV receptor, promoting virus entry into a variety of cell types, and again gB was said to bind PDGFRα (33). We demonstrated that PDGFRα did not function for entry of pentamer-expressing HCMV into epithelial or endothelial cells (34). However, overexpression of PDGFRα apparently altered signaling in epithelial cells, so that there was entry by a pathway not normally utilized in these cells. More recently, the trimer has been shown to bind directly to PDGFRα, and anti-PDGFRα antibodies are able to block HCMV lab strains (that lack the pentamer) from entry into fibroblasts (26). Other laboratories have also shown trimer-specific entry into fibroblasts involving PDGFRα, and several of these reports showed that PDGFRα does not mediate entry into epithelial and endothelial cells (35, 36). The surface molecule THY-1 (CD90) was also described as a receptor for HCMV as it binds to both gB and gH/gL and promotes entry of both lab-adapted and clinical isolates of HCMV (37), and THY-1 apparently affects cell signaling that promotes macropinocytosis of virus (38). It is important to note that all of the entry mediators described above can explain entry of lab-adapted HCMV strains that lack the pentamer. Therefore, observations that pentamer is required for entry into epithelial and endothelial cells and monocyte-macrophages, coupled with our interference data, strongly argue for additional entry mediators that depend upon the pentamer in these cell types.

In this report, we screened a cDNA library obtained from epithelial cells and identified a novel molecule, CD147, which promotes HCMV entry into epithelial and endothelial cells. CD147 expression specially promoted entry of pentamer-expressing HCMV and not virus lacking the pentamer. CD147 antibodies and shRNAs blocked HCMV entry into epithelial and endothelial cells, and virus particles were colocalized with CD147 on cell surfaces and in endosomes. However, soluble CD147 did not block HCMV entry, suggesting that the effects of CD147 are indirect, e.g., CD147 increases the cell surface or endosomal localization of other proteins with which HCMV interacts during entry. CD147 is the first HCMV entry mediator to be described that promotes virus entry via the pentamer into epithelial and endothelial cells.

## RESULTS

### Construction of a human ARPE-19 cDNA library and screening for genes that promote HCMV entry.

**FIG 1:**
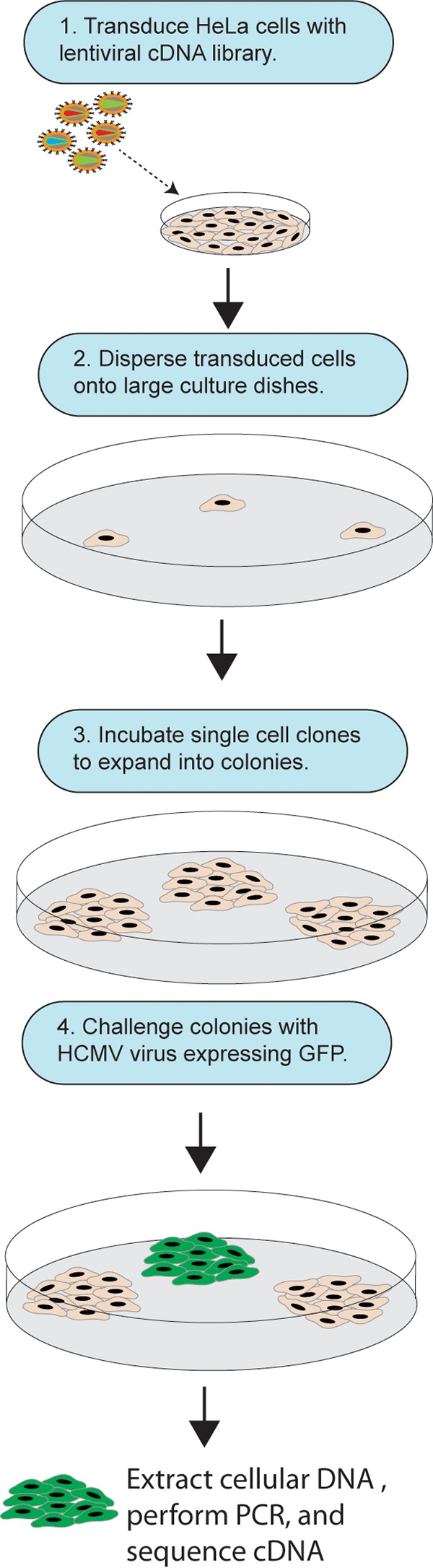
Cartoon depicting cDNA library screening strategy. A lentivirus-based cDNA library derived from ARPE-19 cells was transduced into HeLa cells. The HeLa cells were then trypsinized and dispersed into large culture dishes (~30,000 cells per 150-cm^2^ tissue culture dish) so that colonies derived from single cells grew over 10 days. After colonies of several hundred cells developed, the colonies were challenged with BAD*r*UL131, an HCMV recombinant expressing the pentamer and a GFP reporter gene. At 48 h after HCMV challenge, GFP-positive clones were removed from dishes by using a Pasteur pipette, and the genomic DNA was extracted, purified, and used for PCR followed by sequencing of the amplified cDNA inserts.

**FIG 2:**
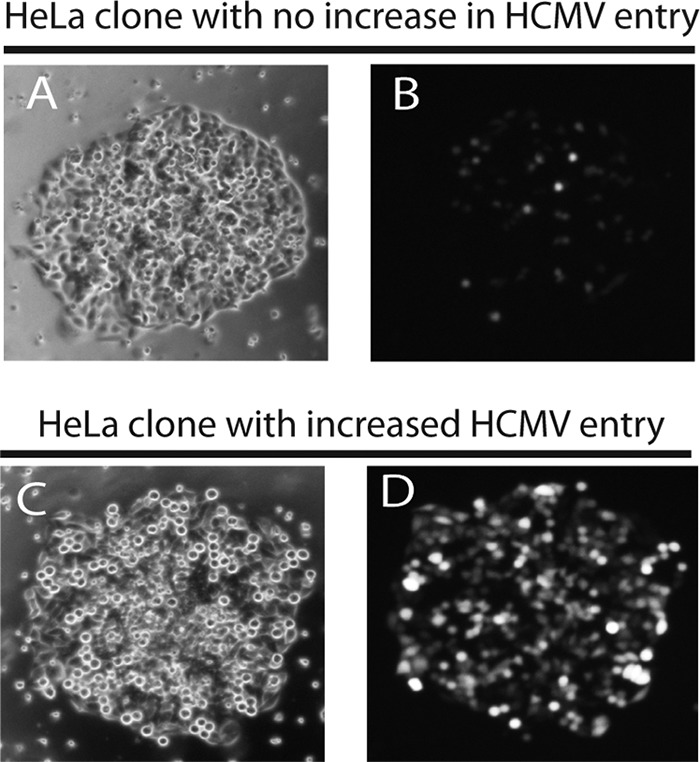
cDNA-expressing HeLa clones showed increased entry for HCMV. Colonies of HeLa cells expressing different ARPE-19 cDNAs (see Fig. 1) were challenged with BAD*r*UL131, which expresses the pentamer complex and GFP. (A and B) Bright-field and fluorescent microscopic images, respectively, of a clone that did not display increased GFP and hence displayed low-level HCMV entry, similar to what we observed with normal HeLa cells (data not shown). (C and D) Bright-field and fluorescent microscopic images, respectively, of a clone that showed increased numbers of GFP-positive cells, indicating higher levels of HCMV entry.

There have been few efforts to identify HCMV receptors or entry mediators that were not based on preconceived notions about proteins that promote HCMV entry. Moreover, all the studies reported to date have characterized molecules that promote entry of laboratory strains of HCMV that lack the pentamer. We chose to screen large numbers of human cDNAs derived from ARPE-19 epithelial cells for the capacity to increase entry of pentamer-expressing HCMV. This approach required identifying human, primate, or animal cells that HCMV does not enter. HCMV replicates very poorly or not at all in most transformed or immortalized human or primate cell lines, but most of these cells show some limited levels of HCMV immediate-early (IE) proteins upon incubation with HCMV. Many rodent cells also express detectable IE proteins; however, there are many caveats for attempting to convert these cells to HCMV-susceptible cells by transduction with human genes, because multiple human genes might be necessary. We found that HCMV infection of HeLa cells produced relatively few infected cells (1 to 5%). We constructed a cDNA library derived from ARPE-19 epithelial cells that contained 8.4 × 10^6^ primary clones, indicating that our library was sufficiently representative of even low-level-expressing genes. cDNAs were inserted into vesicular stomatitis virus (VSV) G-protein-pseudotyped lentivirus vectors. To screen for cDNAs that mediate entry of HCMV, we followed the protocol outlined in Fig. 1. HeLa cells were transduced with lentiviruses expressing ARPE-19 cDNAs for 24 h, and then the cells were trypsinized and sparsely plated on larger dishes so they could form individual colonies. The colonies of transduced HeLa cells were then challenged with HCMV BAD*r*UL131 virus particles, a lab strain of HCMV that was repaired so as to express the pentamer and also expresses green fluorescent protein (GFP) (39). Under these conditions, the vast majority of clones showed very little evidence of HCMV entry based on GFP expression. An example of a HeLa cell colony that displayed little GFP is shown in Fig. 2A and B. However, there were very rare clones that displayed a marked increase in HCMV entry. In a given experimental screen, approximately 1 million colonies were challenged with HCMV, and ~100 showed increased levels of GFP expression. An example of a HeLa cell colony that expressed substantially more GFP is shown in Fig. 2C and D. GFP-positive colonies were harvested by using a Pasteur pipette, the genomic DNA was isolated, and lentivirus-derived cDNAs from the cells were sequenced. From the clone shown in Fig. 2C and D, we identified a cDNA sequence that encoded the cell surface molecule CD147, a member of the immunoglobulin superfamily, also known as extracellular matrix metalloproteinase inducer, or basigin (40). There are four different splice variants of CD147, isoforms 1 to 4, but CD147 isoform 2 is the most common splice variant of CD147 and was found in this screen (41). Other GFP-positive colonies were isolated; however, many of these HeLa cell colonies contained cDNAs that encoded proteins that were unlikely to mediate HCMV entry, e.g., transcription factors or nuclear proteins. Many of these cells contained more than a single lentivirus insert, which may account for these observations to some extent.

### Transduction of HeLa cells to overexpress CD147 promotes increased entry of pentamer-expressing HCMV.

**FIG 3:**
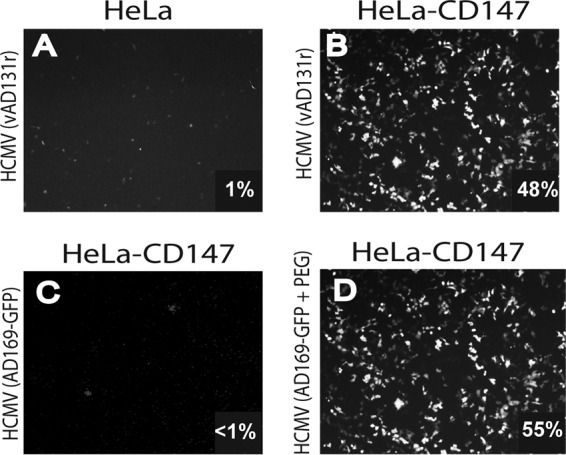
Transduction of HeLa cells with CD147 enhances HCMV entry. (A and B) HeLa cells were transduced with an empty retrovirus (A) or a retrovirus expressing CD147 (HeLa-CD147) (B), propagated in puromycin to select for retrovirus transduction, and then challenged with HCMV BAD*r*UL131, which expresses the pentamer and GFP. (C) HeLa-CD147 cells were challenged with HCMV AD 169, which does not express the pentamer and expresses GFP (AD 169-GFP). (D) HeLa-CD147 cells were challenged with AD 169-GFP for 2 h, and then the cells were treated with a solution of buffered 43% PEG, which promotes entry of virus into cells. The level of HCMV entry is based on the percentage of GFP expression (indicated on the bottom right of each panel).

To examine the effects of CD147 on HCMV entry, an independent CD147 cDNA was obtained from a commercial source (clone ID 38673352, isoform 2; Dharmacon), and this cDNA was expressed by using a retrovirus vector that also expressed a puromycin resistance gene. This retrovirus expressing CD147 and an empty retrovirus were used to transduce HeLa cells, retrovirus-infected cells selected using puromycin, and then the cells were challenged with HCMV BAD*r*UL131. In this experiment, less than 1% of the HeLa cells transduced with empty retrovirus were GFP positive (infected with HCMV), whereas 48% of the CD147-expressing cells were GFP positive (Fig. 3A and B). In other experiments, the infection of CD147-expressing HeLa (HeLa-CD147) cells by BAD*r*UL131 ranged from 30 to 50%. To determine whether pentamer was needed for entry into CD147-expressing HeLa cells, these cells were challenged with a GFP-expressing version of lab-adapted HCMV strain AD169 (AD169-GFP), which does not express the pentamer. GFP expression in these cells was very low, at less than 1% infection (Fig. 3C). To show that replication of AD 169-GFP in HeLa-CD147 cells was blocked at the stage of virus entry, the cells were challenged with AD 169-GFP and briefly treated with polyethylene glycol (PEG), which acts as a chemical fusogen that mediates entry of viruses that are blocked for entry. PEG treatment dramatically increased GFP expression following AD 169-GFP infection of HeLa-CD147 (Fig. 3D). This confirmed that the defects in GFP expression by HCMV lacking the pentamer involved an inability to enter HeLa-CD147 cells. We noted that PEG treatment did not result in GFP expression in 100% of these CD147-HeLa cells; ~55% of the cells expressed GFP, similar to the numbers of GFP^+^ cells when pentamer-expressing virus was used. These results showed that CD147 markedly enhances HCMV entry into HeLa cells, and this entry depends upon the presence of the pentamer.

### CD147 antibodies block entry of HCMV into normally permissive epithelial and endothelial cells.

**FIG 4:**
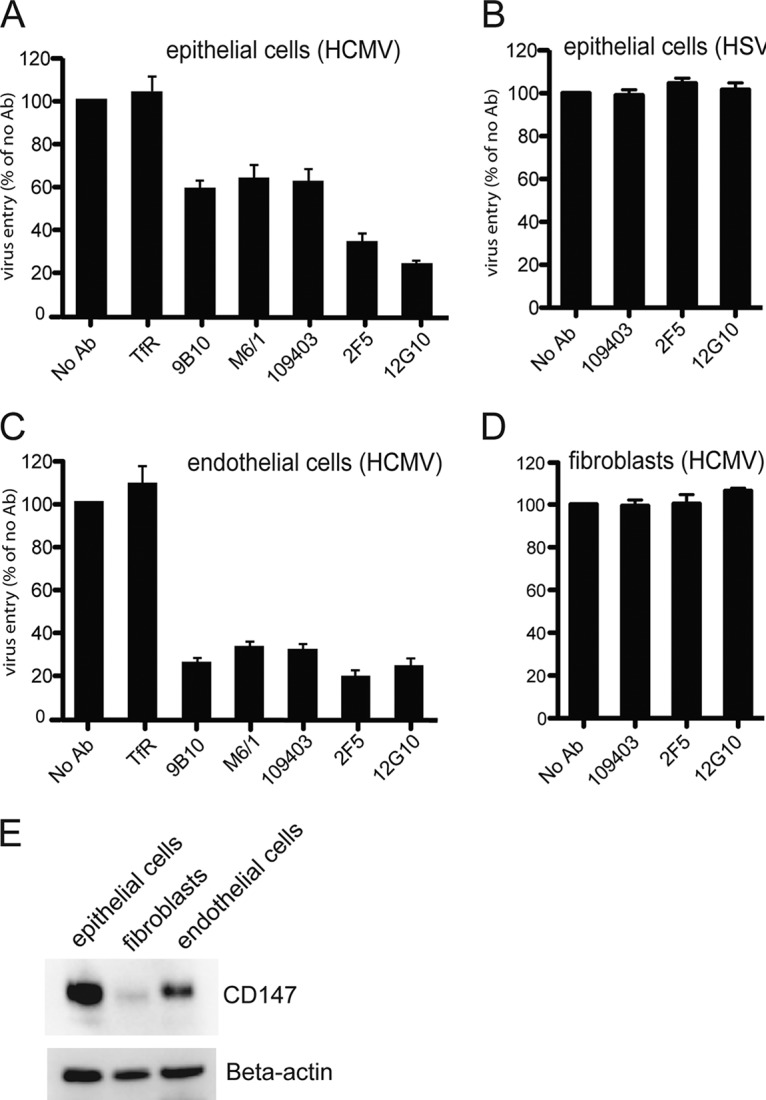
Inhibition of HCMV entry via anti-CD147 antibodies. (A) Human ARPE-19 epithelial cells were left untreated (No Ab) or were pretreated with MAbs specific for the transferrin receptor (TfR) or CD147 (MAbs 9B10, M61, 109403, 2F5, and 12G10) for 1 h at 37°C and then HCMV BAD*r*UL131 was added to these cells in the presence of the MAbs for an additional 2 h at 37°C. The virus inoculum was removed, and the culture medium was replenished with fresh growth medium that contained the MAbs for 24 h, after which HCMV entry was assessed. (B) Human retinal epithelial cells were either left untreated or treated with antibodies as described above and then incubated with HSV-1 F-BAC VP26GFP, an HSV recombinant that express a GFP-tagged tegument protein. (C and D) Antibody inhibition assays were performed as described for panel A with HUVECs or fibroblast cells, respectively, and then challenged with HCMV BAD*r*UL131. Under all experimental conditions, virus entry was quantified by counting GFP-positive from at least three independent wells, and these values compared to the numbers of GFP-positive cells among those not treated with antibodies. (E) Lysates from epithelial, endothelial, or fibroblast cells were analyzed by Western blotting to detect endogenous CD147 by probing membranes with anti-CD147 MAb 109403 or with polyclonal antibodies against beta-actin as a loading control.

Pentamer-expressing HCMV enters epithelial cells and endothelial cells, while HCMV lacking the pentamer enters these cells only poorly (19, 21, 39). These cells are among the most biologically relevant cells that HCMV infects *in vivo*. To determine whether CD147 influences HCMV entry into epithelial and endothelial cells, antibody inhibition experiments were performed. Initially, these experiments involved three commercial monoclonal antibodies (MAbs) specific for CD147, MAbs 9B10 and M6/1 from Abcam, Inc., and MAb 109403 from R&D Systems. However, given that we did not know whether these antibodies possessed the capacities to block CD147 or cause its internalization, we also prepared a soluble form of CD147 (see below) and produced several independently isolated MAbs, including 2F5 and 12G10. All MAbs were purified by using protein A-Sepharose. Human retinal epithelial cells or human endothelial cells were left untreated (NoAb) or were preincubated with a negative-control MAb specific for transferrin receptor (TfR) or with MAbs 9B10, M6/1, 109403, 2F5, or 12G10, using 50 µg/ml for 1 h at 37°C. The cells were then incubated with BAD*r*UL131, an HCMV that expresses pentamer and GFP, in the presence of these antibodies for 2 h, and then the antibodies and virus were removed and the cells were incubated for an additional 24 h in the presence of antibodies before GFP expression was analyzed by fluorescence microscopy. Under these conditions, HCMV entry into epithelial cells was reduced by 40 to 78% by different anti-CD147 MAbs, compared with cultures with no antibodies or the TfR MAb (Fig. 4A). There was no effect of several of these antibodies on the entry into epithelial cells of a herpes simplex virus recombinant expressing a fluorescent capsid protein (Fig. 4B). Anti-CD147 MAbs also inhibited entry of HCMV into endothelial cells more uniformly by as much as 80% (Fig. 4C). Inhibition of HCMV entry into epithelial and endothelial cells with 25 µg/ml was approximately half that observed here with 50 µg/ml (data not shown). These anti-CD147 MAbs did not inhibit entry of HCMV into fibroblasts (Fig. 4D). A Western blot analysis of epithelial, endothelial, and fibroblast cells showed differences in endogenous CD147 expression levels, with epithelial cells expressing the highest amounts, followed by endothelial cells and lower quantities of CD147 expression in fibroblast cells (Fig. 4E). These results with several independently isolated anti-CD147 MAbs showed that CD147 plays an important role in HCMV entry into epithelial and endothelial cells, but not fibroblasts.

### shRNA silencing of CD147 reduces HCMV infection of endothelial cells, but not infection of fibroblasts.

**FIG 5:**
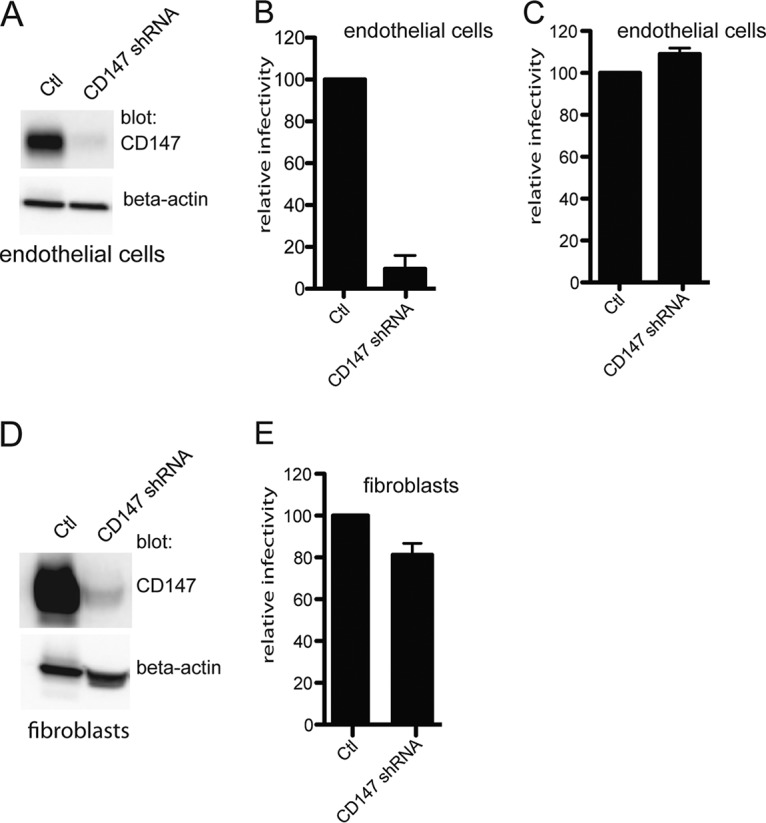
Silencing CD147 inhibits HCMV entry into endothelial cells. (A) Lysates from a control endothelial cell line (tAECs) transduced with an empty lentivirus vector (Ctl) or an endothelial cell line (tAECs) transduced with a lentivirus expressing a CD147-specific shRNA (CD147 shRNA; clone ID V3LHS_412785; Dharmacon) were analyzed in Western blot assays to determine the level of CD147 silencing. Membranes were probed with either anti-CD147 MAb 109403 or a rabbit polyclonal antibody against beta-actin (a loading control). (B) Human endothelial cells (tAECs) were transduced with an empty lentivirus vector (Ctl, lacking any shRNA) or a lentivirus vector expressing an shRNA to CD147 (CD147 shRNA) and then selected by puromycin. The cells lines were then infected with HCMV strain BAD*r*UL131, and the level of entry was assessed by monitoring GFP expression from at least three independent wells. (C) The lentivirus-transduced cell lines in panel B were infected with HSV-1 VP26-GFP and analyzed for GFP expression after 24 h. (D) Lysates from control fibroblasts transduced with an empty lentivirus vectors (Ctl) or CD147 shRNA-expressing fibroblasts (CD147 shRNA) were analyzed by Western blotting to characterize CD147 silencing as described for panel A. (E) Fibroblast cell lines that had been transduced with an empty lentivirus vector (Ctl) or a lentivirus vector expressing an shRNA to CD147 (CD147 shRNA) were established using puromycin and then infected with HCMV BAD*r*UL131, after which the number of GFP-positive cells was analyzed.

To further examine the role of CD147 in HCMV entry, we silenced CD147 by using a lentivirus vector expressing a single CD147-specific shRNA (clone ID V3LHS_412785; Dharmacon). Silencing of CD147 in our retinal epithelial cells resulted in a cell line that was very difficult to propagate, consistent with other observations that CD147 silencing can inhibit epithelial cell proliferation and that CD147 is essential in mice for proper development of the retina (42, 43). However, we successfully established endothelial and fibroblast cell lines with the lentivirus expressing the CD147-specific shRNA, selecting for lentivirus-transduced cells using puromycin. CD147 expression was reduced by 90% in endothelial cells transduced with the lentivirus expressing the CD147-specific shRNA, compared with cells transduced with a lentivirus lacking an shRNA (Fig. 5A). When CD147-silenced endothelial cells were infected with HCMV BAD*r*UL131, the numbers of GFP-positive cells was reduced by >90% compared to the numbers of GFP^+^ cells transduced with the empty lentivirus vector (Fig. 5B). To evaluate potential off-target effects of CD147 silencing, the CD147 shRNA-expressing cells were infected with an herpes simplex virus (HSV) recombinant expressing a fluorescent capsid protein (VP26-GFP). Silencing of CD147 did not reduce HSV-1 entry (Fig. 5C). The antibody inhibition assays described above suggested that CD147 is not important for HCMV entry into fibroblasts. To extend these observations, CD147 was silenced in fibroblasts (Fig. 5D). HCMV was able to enter fibroblasts expressing the CD147-specific shRNA, and there was only a small decrease in GFP^+^ cells, compared with fibroblasts transduced with empty vector (Fig. 5E). These studies provided additional support for the hypothesis that CD147 plays an important role in HCMV entry into endothelial cells but is not important in fibroblasts.

### Effects of a soluble form of CD147 on HCMV entry and binding to soluble HCMV glycoproteins.

**FIG 6:**
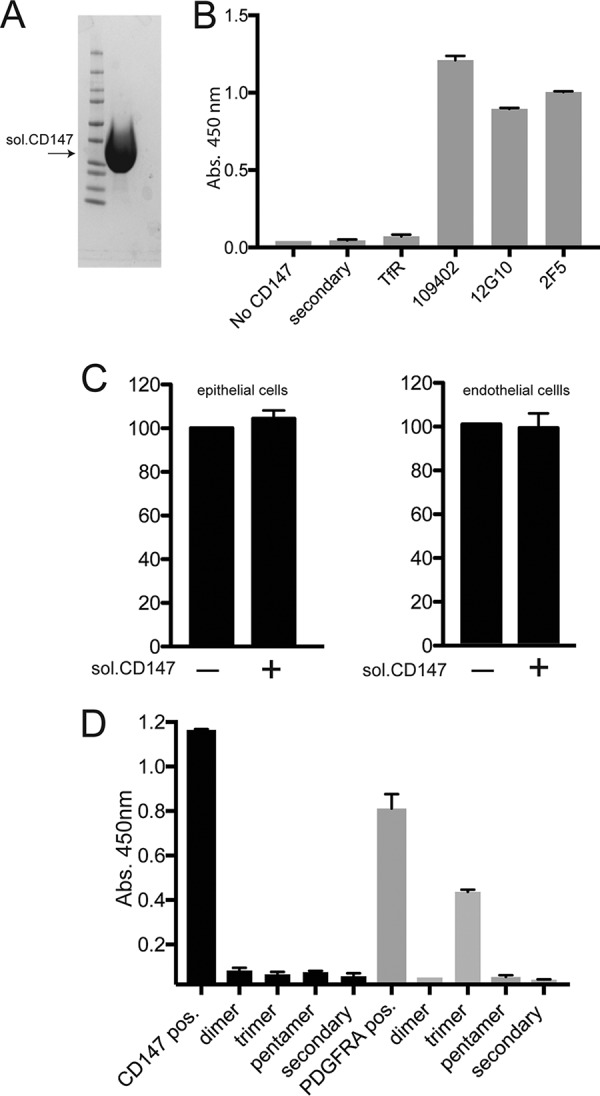
Effects of soluble CD147 on HCMV entry and binding to soluble HCMV glycoproteins. (A) A soluble form of CD147 that was fused to a polyhistidine epitope tag and purified from the tissue culture supernatant of plasmid-transfected 293E cells via use of nickel-agarose was analyzed by electrophoresis, polyacrylamide gel staining, and Coomassie brilliant blue stain. (B) Soluble CD147 was bound to nickel-coated plates and then analyzed in an ELISA. Individual wells were incubated with either a control MAb to transferrin (TfR) or CD147-specific MAbs 109403, 12G10, or 2F5, followed by goat anti-mouse–HRP conjugate. Controls also included incubation with secondary antibodies only (secondary). The bound antibodies were detected by chemiluminescence by adding Turbo-TMB ELISA substrate (Thermo Fisher), and absorbance was read using a precision plate reader (Molecular Dynamics). (C) HCMV BAD*r*UL131 virus particles were incubated with soluble CD147 (sol. CD147^+^) at 100 µg ml^−1^ or no protein (sol. CD147 -) for 1 h at 37°C and then added to ARPE-19 epithelial cells or HUVECs for 2 h at 37°C. The virus inoclula were removed, and cells were incubated an additional 24 h before the numbers of GFP^+^ cells were assessed. Relative infectivity was calculated by comparing the numbers of GFP^+^ cells in soluble CD147 treated groups versus no-protein controls. (D) Soluble CD147 (black bars) or soluble PDGFRα (gray bars) were allowed to adsorb onto microtiter plates and then incubated with soluble gH/gL (dimer), trimer, or pentamer complexes. The plates were washed and then incubated with anti-gH MAb 14-4b, washed, and incubated with goat anti-mouse–HRP conjugate. Positive controls included anti-CD147 or anti-PDGFRα MAbs followed by secondary goat anti-mouse–HRP conjugate (CD147 pos. and PDGFRα pos.). Negative controls involved wells with adsorbed proteins incubated with secondary antibodies only (secondary). Chemiluminescence was detected as described for panel B.

**FIG 7:**
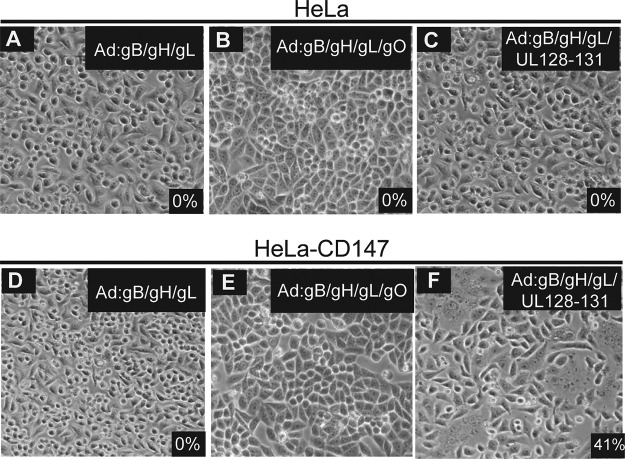
CD147 induces cell-cell fusion of HeLa cells expressing HCMV fusion proteins. (A to C) HeLa cells were transduced with Ad vectors expressing HCMV gB and gH/gL (A), gB and gH/gL/gO (B), or Ad vectors expressing gB, gH/gL, UL128, UL130, and UL131 (C) and assayed for the presence of cell-cell fusion. (D to F) HeLa cells were transduced with a retrovirus to express CD147 and then transduced with Ad vectors expressing HCMV gB and gH/gL (D), gB and gH/gL/gO (E), or Ad vectors expressing gB, gH/gL, UL128, UL130, and UL131 (F) and assayed for the presence of cell-cell fusion. Fusion was quantified by comparing the number of nuclei involved in cell-cell fusion events (involving ≥5 cells per fusion event) with the total number of nuclei.

The capacity of a soluble form of a viral entry mediator to block virus entry provides evidence for direct interactions between viral proteins and the entry mediator. We constructed a soluble form of the extracellular domain of CD147 protein (encompassing amino acids 1 to 204) that was fused at the C terminus to a polyhistidine epitope tag. Soluble protein was purified from the cell culture supernatants of plasmid-transfected 293E cells by using immobilized nickel-nitrilotriacetic acid (NTA)–agarose (Fig. 6A). The protein preparation was free of other protein contaminants, and there was no evidence of proteolysis. We validated the structure of the soluble CD147 by testing whether several MAbs recognized CD47. These anti-CD147 MAbs blocked HCMV entry (Fig. 4) and, therefore, must recognize native CD147. Three different CD147 MAbs bound to soluble CD147 (Fig. 6B). The purified CD147 protein (100 µg ml^−1^) was added to serum-free culture medium that contained HCMV BAD*r*UL131 virus particles and incubated for 1 h at 37°C. The protein-virus mixture was then added to epithelial or endothelial cell monolayers for 2 h 37°C before the soluble protein and virus were removed and replaced with growth medium containing the soluble CD147 (100 µg ml^−1^); entry of HCMV was assessed 24 h later by measuring GFP expression. Under these conditions, there was no detectable inhibition of HCMV BAD*r*UL131 entry into either epithelial or endothelial cells for soluble CD147-treated virus compared to virus treated with no protein (Fig. 6C). These observations suggest that HCMV does not directly interact with CD147. We also characterized whether soluble forms of gH/gL, trimer, or pentamer produced in mammalian 293 cells could bind to soluble CD147. Soluble CD147 or PDGFRα was adsorbed onto enzyme-linked immunosorbent assay (ELISA) plates and then incubated with soluble HCMV gH/gL proteins; binding was assessed by measuring chemiluminescence. The results of these assays showed no evidence that soluble gH/gL, trimer, or pentamer was able to bind to our soluble CD147 (Fig. 7D). In contrast, we observed binding of soluble HCMV trimer to a soluble version of PDGFRα (Fig. 7D).

### CD147 expression increases cell-cell fusion induced by HCMV glycoproteins.

An important method for determining whether a viral entry mediator directly or indirectly affects the viral entry machinery is to test whether the cellular protein increases fusion of cells produced by expressing viral glycoproteins. Increased cell-cell fusion provides evidence that the cellular protein affects the viral fusion machinery rather than altering cells, e.g., by signal transduction. We previously described cell-cell fusion following transduction of cells with gB, which is believed to act directly in fusion, and gH/gL proteins, which are thought to trigger gB for fusion (30). In all the cell types we have tested, expression of gH/gL (without gO or UL128 to -131) along with expression of gB were sufficient for cell-cell fusion. Here, we used nonreplicating adenovirus (Ad) vectors to deliver HCMV gB and gH/gL, gO, or the pentamer (gH/gL/UL128 to -131) into HeLa or HeLa-CD147 cells (HeLa cells transduced with retroviruses to express CD147). Normal HeLa cells did not fuse following adenovirus vector expression of gB and either gH/gL, gH/gLgO, or the pentamer (Fig. 7A to C). Similarly, there was no fusion of HeLa-CD147 cells when gB was expressed with gH/gL or when gH/gL/gO were expressed in the cells (Fig. 7D and E). However, there was extensive fusion of HeLa-CD147 cells when gB and the pentamer were expressed in the HeLa-CD147 cells, with 44% of the cells involved in fusion events (Fig. 7F). These results were surprising and also strongly supported the hypothesis that CD147 acts to promote HCMV entry into cells by promoting the viral fusion machinery, rather than having more indirect effects on cells.

### HCMV virus particles colocalize with CD147 on the surfaces and in endosomes of ARPE-19 epithelial cells.

**FIG 8:**
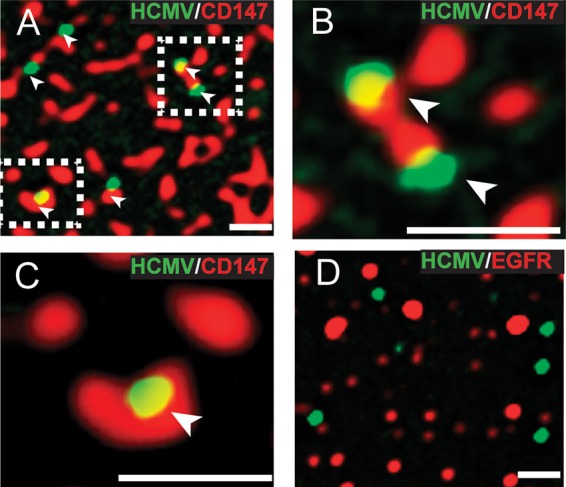
HCMV colocalizes with CD147 patches on the surface of ARPE-19 cells. (A) ARPE-19 cells on glass slides were incubated with anti-CD147 MAb 109403 and HCMV UL32-GFP virus particles at 4° for 1 h, then shifted to 37° for 15 min, and then fixed. The fixed cells were then incubated with goat anti-mouse–594 fluorescent secondary antibody to detect surface CD147 and then the cells were analyzed by deconvolution microscopy. Arrows indicate green fluorescent HCMV virus particles that overlapped with red fluorescent channel signal from CD147 surface patches. (B and C) Magnified images of the squared areas from panel A. (D) ARPE-19 cells were incubated with anti-EGFR MAb (LA1) and HCMV UL32-GFP virus particles at 4°C for 1 h, then shifted to 37°C for 15 min, fixed, and then processed to detect surface EGFR as described for panel A. The scale bars on the bottom right of each panel represent 1 µm.

**FIG 9:**
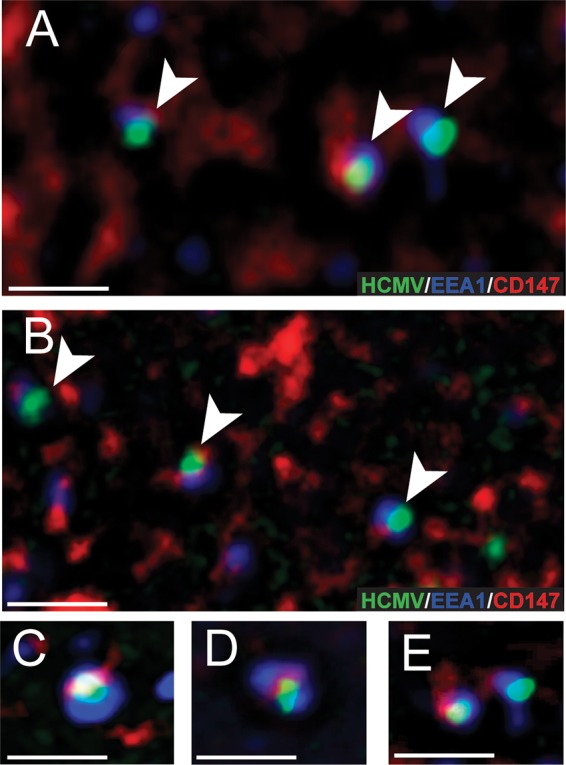
HCMV colocalizes with CD147 in early endosomes. ARPE-19 epithelial cells on glass slides were transduced with a baculovirus expressing a fluorescent-tagged early endosome marker, EEA-1. The cells were then incubated with HCMV UL32-GFP virus particles for 1 h at 4°C and then shifted to 37°C for 1 h. The cells were fixed and processed for immunofluorescent staining with an anti-CD147 MAb (109403) followed by a 649-conjugated secondary antibody to detect CD147 and then analyzed by deconvolution microscopy. (A and B) Representative images with arrows depict HCMV particles (green) colocalizing with CD147 (red) in EEA-1-labeled endosomes (blue). (C to E) More highly magnified images of HCMV particles (green) colocalizing with CD147 (red) and EEA-1-labeled endosomes (blue). The scale bars on the bottom left of each panel represent 1 µm.

Many entry mediators colocalize with virus particles at cell surfaces or in endosomes. Entry of HCMV into ARPE-19 cells involves virus binding to cells surfaces, which triggers macropinocytosis, and later there is fusion between virions and endosomal membranes (30). To examine cell surface localization, we incubated ARPE-19 cells with both an anti-CD147 antibody (MAb 109403) and HCMV TB40E-UL32-GFP recombinant virus particles, which have a GFP-tagged pp150 tegument protein, at 4°C for 1 h (44). Subsequently, the cells were shifted to 37°C for 15 min so that the antibodies promoted patching of cell surface CD147 molecules. The cells were then fixed and incubated with goat anti-mouse fluorescent secondary antibodies to detect CD147 antibodies, and then the cells were analyzed by deconvolution fluorescence microscopy. There were clear associations of cell surface patches of CD147 (red signal) and most HCMV particles (green signal) (Fig. 8A to C). Analysis from 25 different images of GFP-labeled HCMV particles showed 74% of these particles with overlapping fluorescent signal with CD147 patches. In contrast, when a MAb specific to the EGFR was used to patch cell surface EGFR, only 5% of HCMV particles were observed to have overlapping fluorescent signals with EGFR patches (Fig. 8D). To determine whether CD147 was found in the same endosomal compartments as HCMV, we expressed a fluorescent-tagged early endosome marker protein, EEA-1, in ARPE-19 cells by using a recombinant baculovirus vector that expresses VSV-G protein (BacMam). These cells were incubated with HCMV-UL32-GFP virus particles at 4°C for 1 h to allow virus to bind to cell surfaces, and then the cells were shifted to 37°C for 1 h to allow internalization. The cells were then fixed and stained with anti-CD147 MAb followed by DyLite-649 (red signal) conjugated to goat anti-mouse secondary antibody; the stained cells were viewed by deconvolution microscopy. GFP-labeled HCMV virus particles (green signal) were often (~28%) colocalized within EEA1-positive endosomes (blue signal) (Fig. 9A to E). We also observed that 80% of these endosomes containing HCMV virus particles also contained CD147 (red) (Fig. 9A to E).

## DISCUSSION

HCMV can infect many diverse cell types, and there is good evidence that the virus enters different cells by using different mechanisms involving distinct virus glycoproteins. For example, early studies suggested that HCMV enters fibroblasts by fusion of the virus envelope directly with the plasma membrane following adsorption onto heparan sulfate glycosaminoglycans (GAGs) and perhaps by trimer-mediated interactions with cell surface PDGFRα (26, 35, 36, 45). More recent studies suggested that HCMV enters fibroblasts by clathrin-independent, pH-independent macropinocytosis (46). It appears that trimer interactions with PDGFRα are important and perhaps all that is needed for triggering the gB fusion protein in this process (26, 35, 36). In contrast, the pentamer is required in addition to trimer for entry into epithelial cells, a process that likely also requires heparan sulfate GAGs for adsorption followed by macropinocytosis and fusion with endosomes in a process that requires low-pH in the endosome (17, 34). Without pentamer virus particles remain trapped in endosomes for long periods, apparently because gB-mediated entry fusion does not occur (34).

We attempted to identify cellular molecules that are involved in HCMV entry into epithelial cells by screening a cDNA library generated from retinal epithelial cell RNA and introducing the library into HeLa cells by lentivirus transduction. We identified CD147 by this screen; this represents the first example of an HCMV entry mediator derived from an unbiased cDNA library screen. Importantly, we focused on cellular proteins that mediated entry of a wild-type strain of HCMV (i.e., one that expresses the pentamer complex), and CD147 represents the first virus entry mediator that functions to mediate entry by a pathway that requires the pentamer. HeLa cells were chosen for this screen because these cells were mostly refractory to HCMV entry, and being human cells this increased the likelihood that other human coreceptors might be required during entry. However, HeLa cells are tumor cells, HCMV does not replicate well in any tumor cell line, and these cells are not considered biologically relevant in HCMV pathogenesis. It also made little sense to us to transduce other human or primate cell lines with CD147, because our previous studies (30) and other unpublished studies have shown that tumor cell lines are not simply blocked for virus entry but instead are blocked in uncoating and early gene expression. Thus, rather than characterizing how CD147 functions to increase HCMV entry into CD147-HeLa cells, we characterized the effects of anti-CD147 antibodies and shRNAs on virus entry into permissive and biologically relevant cells, such as epithelial and endothelial cells.

A panel of independently isolated anti-CD147 MAbs inhibited HCMV entry into both epithelial and endothelial cells. Importantly, these antibodies had no effect on HCMV entry into fibroblasts. The antibodies also had no effect on entry of HSV into epithelial cells. Coupled with observations that these effects were observed with at least 5 different MAbs, this is strong evidence that HCMV depends upon CD147 expression for entry into epithelial and endothelial cells. Antibodies can function in several ways to block virus entry, including the following: (i) blocking important entry mediators on cell surfaces, (ii) reducing cell surface expression of molecules, or (iii) acting in endosomes to block the entry that is triggered by these molecules. Presently, it is not clear how these antibodies block HCMV entry.

We were unable to silence CD147 in ARPE-19 retinal epithelial cells by using shRNAs, because this was toxic to these cells. It is known that CD147 is essential for the development of the retina (43). However, we successfully produced endothelial and fibroblast cell lines expressing CD147-specific shRNAs that efficiently silenced CD147 expression in these cells. Silencing CD147 in endothelial cells reduced HCMV entry by 90%, while HSV entry into these cells was not affected. In contrast, silencing CD147 in fibroblasts had little effect on HCMV entry. We also showed that CD147 colocalized with HCMV particles on cell surfaces and in endosomes of epithelial cells. Together with the antibody inhibition experiments, these observations are strong evidence that CD147 is important for HCMV entry into endothelial and epithelial cells, but not important for entry into fibroblasts.

Overexpression of CD147 also substantially increased cell-cell fusion when HeLa cells expressed the pentamer complex along with glycoprotein gB. There was no fusion in nontransduced HeLa cells, whereas nearly half the CD147-HeLa cells underwent fusion following expression of gB and pentamer. This is important evidence that CD147 increases virus entry into cells, acting directly or indirectly to promote entry fusion. These observations also argue against CD147-mediated signaling that alters cells so that early viral gene expression is increased. Previous studies of HCMV cell-cell fusion showed that in most cells HCMV gB and gH/gL are sufficient for cell-cell fusion (30, 34). However, here we observed that expression of both pentamer and gB was required for cell-cell fusion involving CD147-HeLa cells. This coincides with observations that CD147-mediated entry into cells requires the pentamer in the virion envelope and strengthens our hypothesis that there is a pentamer-specific step in HCMV entry into epithelial and endothelial cells that CD147 affects. However, it is also possible that the expression of UL128, UL130, and UL131 in HeLa cells increased surface levels of gH/gL in HeLa cells, as we reported previously (23, 47). These cell-cell fusion results are important, because this is the first example of a cellular molecule that can promote the triggering of the HCMV fusion machinery. These findings are in contrast to a previous report that showed that PDGFRα did not increase cell-cell fusion (34).

How CD147 acts to promote entry is not clear, but our observations that soluble CD147 did not inhibit HCMV entry has important implications here. Pat Spear and colleagues coined the term “entry mediator” to describe molecules that promote or enhance the ability of a virus to gain access into a cell in their descriptions of the first two molecules that promoted HSV entry into cells, nectin-1 and HVEM (48, 49). These initial descriptions involved cDNA screens and provided no evidence that specific viral proteins interacted with these proteins, though later it was found that HSV gD interacts with HVEM and nectin-1 (50, 51). Moreover, a third HSV entry mediator was described, acting indirectly as an enzyme that modifies heparan sulfate GAGs and increasing HSV attachment (52). It remains possible that membrane-anchored CD147 plays a postattachment role to regulate endocytic pathways, a scenario that would not be achieved by soluble CD147. However, evidence that CD147 promotes cell-cell fusion argues against alterations in endocytosis, because CD147 must trigger the viral fusion machinery in some manner. It is also formally possible that our soluble CD147 is misfolded or inactive in some manner, though antibodies produced using this protein were able to block entry. A more likely mechanism for how CD147 functions in entry involves increased cell surface or endosomal localization of other proteins. CD147 forms stable complexes with numerous other cell surface proteins, including monocarboxylate transporter (MCT) proteins, C-type lectins, GLUT1, CD44, and CD98, and increases cell surface expression of these proteins (41, 42, 53–55). We expressed a number of these molecules by using retrovirus transduction, including MCT-1, MCT-3, MCT-4, CD44, CD29, CD98, ASCT2, and syndecan-1, but we did not observe increased entry of HCMV into HeLa cells beyond that with CD147 expression alone (data not shown). However, the number of CD147 ligands appears to be high. A recent study involving immunoprecipitation of CD147 followed by mass spectrometry identified 157 potential CD147-interacting proteins (56).

Our hypothesis that CD147 increases HCMV entry by acting indirectly on other cellular proteins needs to be tested in other experiments. However, there are several other examples of these effects. Kaposi’s sarcoma-associated herpesvirus (KSHV) entry is increased by expression of xCT, the 12-transmembrane light chain of the human cystine/glutamate exchange transporter, which was also identified in a cDNA library screen (57). xCT forms a heterodimer complex with the amino acid transporter CD98, which then interacts with several other molecules, including integrins. However, to date, no direct interactions between KSHV proteins and xCT/CD98 have been shown. Interestingly, an early report showed anti-xCT antibodies could block KSHV entry (57). Subsequently, it was determined that xCT antibodies did not block entry but reduced virus gene expression by abrogating the formation of a multiprotein complex consisting of xCT/CD98 and integrins that is required for early virus gene expression (58). The entry of hepatitis C virus (HCV) requires the membrane proteins CD81 and the tight junction protein claudin-1. In this case, the HCV envelope glycoprotein E2 binds directly to CD81 during virus entry; however, subsequent interactions between CD81 and claudin-1 are necessary for proper internalization of virus particles into endosomes (59). These examples highlight the complexity of virus entry pathways, which often involve more than one single entry mediator, especially when endosomal entry pathways are involved. CD147 may be an important clue in efforts to identify other HCMV entry mediators.

## MATERIALS AND METHODS

### Cells and viruses.

Primary human neonatal dermal fibroblasts (NHDFs) obtained from Invitrogen, 293T cells (ATCC), and HeLa cells (ATCC) were all grown in Dulbecco’s modified Eagle’s medium (DMEM) with 10% fetal bovine serum (FBS). Human umbilical cord vascular endothelial cells (HUVECs) and transformed aortic endothelial cells (tAEC) were kind gifts from Ashlee Moses at the Vaccine and Gene Therapy Institute in Portland, Oregon, and were maintained in Medium-200 plus low-serum growth supplement (Invitrogen). Human retinal pigmented epithelial (ARPE-19) cells were obtained from ATCC and grown in DMEM–F-12 plus 10% FBS. All cells were maintained at 37°C with 5% CO_2_. HCMV BAD*r*UL131 (kindly provided by Tom Shenk, Princeton University) is a derivative of AD 169 that has had the UL131 gene repaired to allow for expression of pentamer and also encodes a GFP reporter gene under control of the simian virus 40 (SV40) promoter (39). The HCMV recombinant AD 169-GFP (provided by Tom Shenk, Princeton University) was derived from the laboratory strain AD 169 by engineering a GFP/ SV40 promoter cassette into the virus and does not express the pentamer (39). HCMV TB40E UL32-GFP is a derivative of the endothelial-tropic strain TB40E that has a GFP reporter gene fused to the tegument phosphoprotein (pp150) encoded by the UL32 open reading frame (44). HCMV stocks were produced from NHDFs grown in roller bottles, and viral particles were concentrated from culture supernatants by centrifugation through a cushion of 20% sorbitol in phosphate-buffered saline (PBS) at 80,000 × *g* for 1 h. Pellets were suspended in DMEM plus 10% FBS and frozen at −70°C. Because HCMV does not plaque very well, we determined titers of our HCMV stocks by determining the number of infectious units (IU) per milliliter via serial dilution of virus stocks and then adding dilutions onto NHDF monolayers and staining for the HCMV immediate-early gene IE-86 after 24 with anti-IE-86 rabbit polyclonal serum 6658 (34). HSV-1 F-VP26-GFP has been described previously and was propagated and its titer was determined on Vero cells (60).

### Retroviruses and lentiviruses.

A retrovirus expressing CD147 was generated by cloning the CD147 gene from plasmid pCMV-SPORT6 (clone ID 38673352; Dharmacon) into the retrovirus plasmid pCMMP-IRES-Puro (a gift from Bill Sugden, Addgene plasmid number 36952). A GIPZ lentivirus proviral plasmid expressing an shRNA targeting CD147 was obtained from Dharmacon (clone ID V3LHS_412785). A non-shRNA-expressing GIPZ lentivirus plasmid was used as a control (plasmid RHS4349; Dharmacon). Retrovirus or lentivirus stocks were generated by cotransfecting lentivirus or retrovirus proviral plasmids along with plasmids expressing the appropriate lentivirus or retrovirus GAG/POL/REV genes and a plasmid carrying the VSV-G protein gene into 293T cells by calcium phosphate transfection. At 48 h posttransfection, virus-containing cell supernatants were collected and stored at −70°C. Transduction of cells involved incubation of cell monolayers with supernatant stocks and spinoculating the cells at 2,000 × *g* for 2 h. For selection of cell lines, cell monolayers were incubated in culture medium containing 2 µg ml^−1^ of puromycin at 48 h posttransduction. The surviving cells were then amplified under normal growth conditions with medium containing 2 µg ml^−1^ of puromycin until assays were performed.

### Construction of ARPE-19 cDNA library and library screening.

Poly(A) RNA was extracted from ARPE-19 cells by using the Dynabeads oligo(dT) kit (Invitrogen). cDNAs were synthesized using the Cloneminer II cDNA synthesis kit (Invitrogen) according to the manufacturer’s instructions. The synthesized cDNAs were inserted into the PV1 lentivirus proviral plasmid harboring lentivirus sequences (61) (kindly provided by Charlie Rice, Rockefeller University) that were modified to contain the Gateway-compatible attP1 and attP2 recombination sites, ccdB suicide gene, and the chloramphenicol resistance marker from plasmid pDONR 222 (Invitrogen). Insertion of these cDNAs into modified PV1 plasmids involved using standard Invitrogen Gateway protocols with BP-clonase. Transformation of plasmid DNAs into ElectroMax DH10B bacteria (Invitrogen) involved a BioRad gene pulse electroporator, and bacteria were selected on LB agar plates with chloramphenicol (30 µg ml^−1^). The lentivirus cDNA library exhibited a titer of ~8.4 × 10^6^ primary clones, based on the number of bacterial colonies on LB agar plates after serial dilution. Lentivirus plasmid DNA was isolated from 25 individual colonies analyzed for cDNA insertion by restriction digestion, and cDNA inserts ranged from 0.5 to 3.0 kbp with an average length of 1.8 kbp. The primary cDNA library was amplified by scraping the bacterial colonies into LB agar and then spreading these bacteria onto 200 150-cm^2^ LB agar plates and incubating the bacteria overnight at 37°C. Bacterial colonies were then scraped from the plates and pooled, and the plasmid DNA was isolated using Qiagen columns, aliquoted, and stored at −80°C. The PV1 plasmid library DNA was then used to transfect 293T cells along with plasmids carrying genes for HIV GAG-POL-REV and VSV-G protein, producing VSV-G protein-pseudotyped lentiviral particles that were collected from the culture supernatants after 48 h. Titers were determined by serially diluting lentiviruses and infecting 293T cells followed by immunofluorescent staining for the HIV REV protein with anti-Rev antibody. MAb 1G7 (NIH AIDS Reagent Program) 48 h after transduction. These lentiviruses from 293T cells were added to ~1 × 10^6^ HeLa cells seeded as monolayers in 6-well dishes at ~1 transducing unit per cell, and the transduction was enhanced by centrifuging the dishes in a swinging bucket rotor at 2,000 × *g* for 2 h. The cells were incubated for 24 h and then trypsinized and transferred to 150-cm^2^ tissue culture dishes at 30,000 cells per dish. Approximately 10 days later, the HeLa cell colonies on these dishes were infected with HCMV BAD*r*UL131, and GFP expression was determined by fluorescence microscopy 48 h later. Colonies that showed increased levels of HCMV entry were dislodged from the culture dish by using a Pasture pipette, the genomic DNA was purified using the Gentra Puregene tissue kit, and the genomic DNA was subjected to PCR using primers 5′-CCCAGTCACGACGTTGTAAAACG-3′ and 5′-GAGCGGATAACAATTTCACACAGG-3′, which anneal to proviral DNA sequences that flank the cDNA insertion site. These PCR products were sequenced to identify ARPE-19 cDNA inserts present in the HeLa cells.

### Replication-defective adenovirus vectors.

The construction of nonreplicating (E1 minus) Ad vectors expressing HCMV strain TR glycoproteins, gH, gL, UL128, UL130, and UL131, and gB have been described previously (30, 47, 62). Ad vector-containing stocks were produced by infecting 293 M (Microbix) or 911 (provided by A. J. Van der Eb, University of Leiden, Netherlands) cells at 0.1 PFU/cell. Cells were harvested 6 to 10 days after infection and centrifuged at 2,000 × *g* for 5 min. Cell pellets were suspended in DMEM plus 10% FBS and then sonicated to release cell-associated virus, followed by centrifugation at 5,000 × *g* for 5 min to remove large cellular debris. Virus-containing cell lysates were stored at −80°C. Ad titers were determined by plaque assays on 911 cells.

### Antibodies.

The CD147-specific MAbs 9B10 and M6/1 were purchased from Abcam, Inc. The CD147 MAb 109403 was purchased from R&D Systems. The anti-EGFR MAb LA1 was obtained from Chemicon International. The rabbit polyclonal anti-human beta-actin antibody (C-2206) was obtained from Sigma-Aldrich. The anti-transferrin receptor MAb (H68.4) was obtained from Thermo Fisher. The CD147 MAbs 2F5 and 12G10 were generated at the OHSU Monoclonal Antibody Core from mice that were immunized with a soluble version of CD147. The MAb IgG was purified from hybridoma supernatants using protein A-agarose, eluted with gentle antibody/antigen gentle elution buffer (Pierce), and then desalted using Zeba desalting spin columns (Pierce) equilibrated with Tris-saline.

### Expression and purification of soluble CD147 and PDGFR.

A soluble version (amino acids 1 to 204) of CD147 isoform 2, which included a C-terminal eight-histidine epitope tag, was constructed using PCR with oligonucleotide primers 5′-ATCGCGGCCGCTCAGTGGTGGTGGTGGTGGTGGTGGTGGCTGCGCACGCGGAGCG-3′ and 5′-GATCAAGCTTATGGCGGCTGCGCTGTTCGT-3′ and CD147-pCMV-SPORT6 (clone ID 38673352; Dharmacon), and then the PCR product was inserted into the pTT5 plasmid, which has a CMV promoter, an OriP binding site, and an ampicillin resistance marker (63). Plasmid DNA was purified using a Qiagen column, and the DNA was then used to transfect 293E cells grown in shaker flasks (63). DNA was transfected into cells seeded at a density of 1 × 10^6^ cells ml^−1^ using a concentration of DNA at 2 µg ml^−1^. DNA was transfected by mixing a stock concentration of polyethylenimine (PEI) dissolved in PBS with DNA in culture medium equal to 1/10 of the volume of the cell culture to be transfected at DNA to a PEI ratio (weight/weight) of 1:2. The DNA/PEI mixture was allowed to incubate at room temperature for 2 min and then added directly to cells. At 6 days posttransfection, the cell culture supernatant was collected, filtered through a 0.2-µm membrane (Millipore), and then flowed over a nickel-NTA–agarose (Qiagen) at a flow rate of 0.5 ml/h. The resin was washed with 10 bed volumes of PBS supplemented with 10 mM imidazole, and the protein was eluted with PBS supplemented with 300 mM imidazole. Fractions were collected, desalted using a Zeba desalting spin column (Pierce) that was equilibrated with PBS, and analyzed by PAGE followed by Coomassie staining. A soluble form of PDGFRα was expressed using a DNA fragment corresponding to amino acids 1 to 524 was PCR amplified with oligos 5′-GATCGGGTTTAAACGGTCCTTGGAAGTACAGGTTTTCTTCAGAACGCAGGGTGGGAGCCACCAGCTTCAGC-3′ and 5′-GATCAAGCTTATGGGGACTTCCCATCC-3′ to generate a TEV protease cleavage site at the C terminus and then cloned into a pcDNA 3.0 plasmid with an open reading frame encoding a human Fc domain. The DNA encoding the PDGFRα-Fc fusion was then reamplified with oligos 5′-TAATACGACTCACTATAGGG-3′ and 5′-TAGAAGGCACAGTCGAGG-3′ and cloned into plasmid pTT5. Plasmid DNA was transfected into 293E cells as described above. Soluble PDGFRα was purified by adsorbing the protein from tissue cell culture supernatant onto protein-A agarose, followed by washing with Tris-saline followed by TEV protease buffer (50 mM Tris-HCl [pH 8.0], 150 mM NaCl, and 0.5 mM EDTA) and then treating the protein A-agarose with TEV protease with a polyhistidine tag (50 ng/ml) at room temperature with agitation for 2 h. The protein released from the protein A beads was then incubated with nickel-agarose to remove the TEV protease.

### Expression and purification of soluble gH/gL, trimer, and pentamer.

A plasmid encoding gH (amino acids 1 to 692) fused to a C-terminal polyhistidine tag and gL (without a tag) was constructed. Other plasmids encoding gO or all of UL128, UL130, and UL131 were constructed. Various combinations of these plasmids were cotransfected into 293E as described above, and cell culture supernatants were harvested. The gH/gL proteins were purified using nickel-agarose and concentrated as described above. The details of the construction of these plasmids, expression, purification, and the purity determination (size exclusion chromatography and other analyses) of the soluble forms of gH/gL, trimer, and pentamer will be described in another paper (Jing Liu, Theodore Jardetsky, David C. Johnson, and Adam Vanarsdall, submitted for publication).

### Immunofluorescent staining.

For fluorescent staining, cells on glass slides were fixed with PBS containing 4% formaldehyde for 10 min at room temperature. If necessary, cells were permeabilized with immunofluorescence buffer (IF; 0.5% Triton X-100, 0.5% deoxycholate, 2% bovine serum albumin, and 0.05% sodium azide in phosphate-buffered saline) for 30 min and then incubated with primary antibodies diluted in IF buffer for 1 h. Cells were washed several times with IF buffer and stained with secondary antibodies in IF buffer for 1 h, then mounted on glass coverslips with Fluoromount-G (Southern Biotech) and analyzed using a Deltavision Core DV wide-field deconvolution system in the OHSU Advanced Light Microscopy Core.

### Cell-cell fusion assays.

Cell-cell fusion assays were performed with HeLa cell lines that were produced by transducing cells with an empty retrovirus vector or a retrovirus vector expressing CD147 for 2 days, and then transduced cells were selected for 3 days in the presence of puromycin at 2 µg/ml. These cells were subsequently transduced with nonreplicating Ad vectors expressing HCMV glycoproteins (gB, gH, gL, gO, UL128, UL130, and UL131) at 50 PFU per cell. Cells were concurrently transduced with an Ad vector supplying a tetracycline transactivator protein that activates the promoter driving HCMV protein expression, using 1/5 the total amount of Ad vectors added. Cell-cell fusion was then assessed 24 h later. For quantitative analysis of cell-cell fusion, cells were fixed in 4% paraformaldehyde and analyzed under a bright-field microscope. Cell-cell fusion was quantified by counting the total number of nuclei involved in syncytium formation (5 or more nuclei per polykaryocyte) compared to the total number of nuclei in the field and expressed as the percentage of cells fused.

### HCMV entry assays.

For HCMV entry assays, cells were seeded on 24-well dishes and then incubated with HCMV for 2 h. The virus was then removed, the wells were washed once, and then the growth medium was replaced. HeLa, ARPE-19, and endothelial cells were infected with HCMV using 10 IU per cell (as described above), while fibroblasts were infected with 1 IU/cell. HSV entry assays involved 1 PFU/cell. Antibody inhibition assays involved incubating MAbs (50 µg ml^−1^) with cells for 1 h before adding HCMV and incubating cells for an additional 2 h in the presence of antibodies; then, the inoculum was removed and replaced with fresh growth medium containing antibodies. Soluble CD147 inhibition assays involved soluble CD147 (100 µg ml^−1^) that was incubated with HCMV BAD*r*UL131 at 37°C for 1 h prior to adding the virus and soluble CD147 to cells. In all entry assays, virus entry was determined by counting GFP-positive cells after 24 h of HCMV infection or 12 h after HSV infection and then capturing fluorescent images of cells from three separate wells and counting the total number of GFP^+^ cells and the total number of cells stained with DAPI (4′,6-diamidino-2-phenylindole).

### Colocalization of HCMV and CD147 in cells.

CD147 or EGFR molecules on the surfaces of ARPE-19 cells were patched or aggregated by first incubating cells on glass coverslides with anti-CD147 MAb 109403 or anti-EGFR LA1 (10 µg ml^−1^) and also TB40E UL32-GFP virus particles for 1 h at 4°C, followed by shifting the cells to 37°C for 15 min. The cells were fixed with 4% paraformaldehyde, washed, and then incubated in PBS containing a DyLight goat anti-mouse 594–congugated secondary antibody. For early endosomal localization, ARPE-19 cells on glass coverslides were transduced with a baculovirus vector expressing the early endosome marker EEA1-RFP BacMam 2.0 (CellLight; Invitrogen) for 24 h. The cells were then incubated with HCMV TB40E UL32-GFP virus particles at 4°C for 1 h and then shifted to 37°C for 1 h and then fixed in 4% paraformaldehyde. The cells were then permeabilized in IF buffer, immunostained with anti-CD147 MAb 109403 (1:1,000) and then by a DyLight goat anti-mouse 649–conjugated secondary antibody (1:1,000).

### Enzyme-linked immunofluorescence assay.

For antibody binding studies, soluble CD147 (2 µg/ml in PBS) was added to nickel-coated microtiter plates and allowed to incubate overnight at 4°C. Wells were then washed several times in ELISA wash buffer (50 mM Tris-HCl [pH 7.4], 150 mM NaCl, 2% BSA, and 0.05% Tween 20). Primary antibodies were diluted to 2 µg/ml in ELISA wash buffer added to the wells, and the mixtures were allowed to incubate at room temperature for 1 h followed by several washes. Goat anti-mouse–horseradish peroxidase (HRP) was diluted 1:10,000 in ELISA buffer and added to the wells, the mixture was incubated at room temperature for 1 h. For studies of the binding of HCMV gH/gL proteins to CD147 or PDGFRα, soluble CD147 or PDGFRα was diluted to 2 µg/ml in ELISA binding buffer (100 mM carbonate/bicarbonate [pH 9]) and adsorbed onto Maxisorb microtiter plates (Nunc) overnight at 4°C. Plates were washed several times in ELISA wash buffer and then incubated with soluble gH/gL, trimer, or pentamer complexes diluted to 2 µg/ml in ELISA wash buffer and incubated at room temperature for 1 h. Wells were washed and then incubated with primary MAb 109403 or MAb 35248 (R&D Systems) specific to CD147 and PDGFRα, respectively, or MAb 14-4b specific for gH, and incubated for 1 h at room temperature. Wells were washed and then incubated with a goat anti-mouse–HRP–conjugated secondary antibody diluted 1:10,000 in ELISA wash buffer and for 1 h at room temperature. Negative controls involved wells with adsorbed CD147 or and PDGFRα that were incubated with secondary antibody only. In both cases, microtiter plates were subjected to several final washes, and 100 µl of Turbo-TMB substrate was added to the wells and incubated at room temperature. The chemiluminescent reaction was stopped by adding 100 µl of 1 M sulfuric acid to the wells, and the microtiter plate was analyzed using a Molecular Dynamics precision plate reader set at a wavelength of 450 nm.

### SDS-PAGE and Western blotting.

Cell extracts were prepared by scraping cells into 1 ml PBS, centrifuging the cells at 800 × *g* for 2 min, lysing the cell pellet in protein extraction buffer (10 mM Tris-HCl [pH 7.4], 5 mM EDTA, 150 mM NaCl, 1% Triton X-100, 1 mM phenylmethylsulfonyl fluoride), and then centrifuging for 5 min at 13,000 × *g*. The solubilized fraction was diluted in 2× SDS gel loading buffer, boiled for 5 min, and then samples were loaded onto SDS-polyacrylamide gels and separated by electrophoresis. Proteins were electrophoretically transferred to Immobilon membranes (Millipore) in a buffer containing 25 mM Tris, 192 mM glycine, and 20% methanol. Membranes were blocked in TBST (Tris-buffered saline with 0.1% Tween 20 and 5% nonfat milk) for 30 min and then incubated in TBST containing anti-CD147 MAb 109403 or anti-beta-actin polyclonal (Sigma-Aldrich) at 1:1,000 dilution and allowed to incubate at 4°C overnight. The blots were then washed three times with TBST and incubated for 1 h in TBST with a 1:1,000 dilution of horseradish peroxidase-conjugated goat anti-mouse IgG (Jackson). The blots were washed with TBST, and proteins were visualized by incubating membranes in ECL chemiluminescent detection reagent (Pierce) and then exposing the membranes in an ImageQuant LAS 4000 detection system (General Electric).
